# The effect of ambient temperature on in-hospital mortality: a study in Nanjing, China

**DOI:** 10.1038/s41598-022-10395-6

**Published:** 2022-04-15

**Authors:** Haiping Yu, Wenqi Sheng, Ting Tian, Xianzhen Peng, Wang Ma, Wen Gao

**Affiliations:** 1 Business School of Hohai University, Nanjing, 211100 Jiangsu China; 2 Medical Alliance Department, First Affiliated Hospital of Nanjing Medical University, Nanjing, 210029 Jiangsu China; 3 Scientific and Technical Department, First Affiliated Hospital of Nanjing Medical University, Nanjing, 210029 China; 4 Institute of Food Safety and Assessment, Jiangsu Provincial Center for Disease Control and Prevention, Nanjing, 210009 China; 5 Department of Public Health and Preventive Medicine, Kangda College of Nanjing Medical University, Lianyungang, 222000 Jiangsu China; 6 Medical Department, First Affiliated Hospital of Nanjing Medical University, Nanjing, 210029 Jiangsu China; 7 Oncology Department, First Affiliated Hospital of Nanjing Medical University, Nanjing, 210029 Jiangsu China

**Keywords:** Health occupations, Medical research, Risk factors

## Abstract

To reduce the inpatient mortality and improve the quality of hospital management, we explore the relationship between temperatures and in-hospital mortality in a large sample across 10 years in Nanjing, Jiangsu. We collected 10 years’ data on patient deaths from a large research hospital. Distributed lag non-linear model (DLNM) was used to find the association between daily mean temperatures and in-hospital mortality. A total of 6160 in-hospital deaths were documented. Overall, peak RR appeared at 8 °C, with the range of 1 to 20 °C having a significantly high mortality risk. In the elderly (age ≥ 65 years), peak RR appeared at 5 °C, with range − 3 to 21 °C having a significantly high mortality risk. In males, peak RR appeared at 8 °C, with the range 0 to 24 °C having a significantly high mortality risk. Moderate cold (define as 2.5th percentile of daily mean temperatures to the MT), not extreme temperatures (≤ 2.5th percentile or ≥ 97.5th percentile of daily mean temperatures), increased the risk of death in hospital patients, especially in elderly and male in-hospital patients.

## Introduction

Climate as an influential factor of fluctuation in mortality has been paid more and more attention^1,2^. In recent years, many studies suggest that extreme temperatures may cause negatively affect health and increase the mortality risk of many diseases^3^. Rising or lowering temperature is related to the risk of heat-related illness. Gasparrini et al. collected data from 384 locations for quantifying the total mortality burden attributable to non-optimum ambient temperatures, and the relative contributions from moderate and extreme temperatures^1^. In their study, the temperature was responsible for advancing a substantial fraction of deaths, which corresponds to 7.71% of the mortality in selected countries. Chen et al. reported that cold weather generally increased emergency hospital admissions, especially for respiratory diseases and the elderly population^4^. A similar result by Luo et al. is that cold temperatures would impact stroke admissions in male and youth subjects. Besides, exposure to extreme cold was associated with increased hospitalizations for ischemic and hemorrhagic strokes^5^. Some similar studies in the Korean population showed that both high and low temperatures could increase the risk of hospitalization^6–9^.

This temperature-disease correlation has also been reported in China^10–12^. Literature reports that injuries, nervous, circulatory, and respiratory diseases are sensitive to heat, with the attributable fraction accounting for 6.5%, 4.2%, 3.9%, and 1.85%, respectively. Respiratory and circulatory diseases are sensitive to cold temperatures, with the attributable fraction accounting for 13.3% and 11.8%, respectively^10^. In addition, it has been reported that both cold and hot temperatures increase mortality risk and the relationship varies geographically, and among groups of people^10,11,13^. Compared with North China, South China had a higher minimum mortality temperature, and there was a more pronounced cold effect in southern parts of China and a more pronounced hot effect in northern parts^11^. Deng et al. reported that the elderly (≥ 65 years) are more susceptible to daily mean temperature and diurnal temperature range, and females are more susceptible to high diurnal temperature range (DTR) effect than males^13^.

However, as far as we know, most studies have looked only at the relationship between temperatures and mortality in the general population but not looked at specific groups, such as hospitalized patients. If there is a relationship between temperatures change and inpatient death, finding the regularity can provide a scientific basis for the hospital to reduce in-hospital mortality and improve hospital management quality. In recent years, distributed lag non-linear model (DLNM) has been used to study meteorological conditions and health effects, which is a modeling framework to flexibly describe associations showing potentially non-linear and delayed effects in time series data^1,12,14^. Thus, in this study, we investigated the impacts of ambient temperatures on in-hospital mortality in 10 years by DLNM.

## Methods

### Data collection

Nanjing, the provincial capital of Jiangsu, is an international metropolis with a population of over 9 million residents^15^. It is located in the east of China, on the Yangtze River and near the sea (31°14″ to 32°37″ north latitude, 118°22″ to 119°14″ east longitude) with a humid north subtropical climate. It has four distinct seasons, abundant rain, short spring and autumn, long winter and summer, and the difference between winter and summer temperatures is obvious^16^.

In this study, we collected daily meteorological data of Nanjing of the period ranging from January 1, 2010 to June 30, 2020 from the China Meteorological Data Service Center^17^, including daily mean temperature (Temp, °C), average air pressure (Ap, kPa), and average daily relative humidity (Rh, %). Mortality data included time of death, age, and gender of the patients that died from January 1, 2010 through June 30, 2020 were extracted from the medical records front page system of Jiangsu Province Hospital in Nanjing, Jiangsu. This hospital has a construction area of 410,000 m^2^, 4600 beds, and more than 6500 employees; it receives 10,000 to 20,000 outpatients every day^18,19^.

### Statistical analysis

The relationship between meteorological factors and in-hospital mortality is nonlinear. Thus, a distributed lag non-linear model (DLNM) was used to investigate the potential exposure-lag response association between in-hospital mortality and daily average temperatures. To achieve our research purposes, Quasi-likelihood Poisson in generalized linear modeling (GLM) was used to model the natural logarithm of everyday in-hospital mortality counts. The statistical model was as follows:

$$ {\text{Log}}[{\text{E}}({\text{Y}}_{\text{t}})] = \alpha + cb(Tmean,\, lag) + {\text{ns(time, df)}} + {\text{ns}}({\text{Ap}}_{\text{t}}, {\text{df)}} + {\text{ns}}({\text{Rh}}_{\text{t}}^{\prime}, df) + \beta {\text{DOW}}_{\text{t}} $$

In this model, Ap, average daily Rh, and day of the week (DOW) were also taken in the model as a categorical variable. *E*(*Y*_t_) meant the expected daily counts of in-hospital mortality from January 1, 2010 to June 30, 2020. The cross-basis matrix of temperature [cb(Tmean)] was used to explore the daily mean temperature cumulative and delayed effects^1,20^. A cross-basis can be described as a bi-dimensional space of functions describing simultaneously the shape of the relationship along temperature and its distributed lag effects. Choosing a cross-basis amounts to choose two sets of basis functions, which will be combined to generate the cross-basis functions^14,21^. We used natural cubic spline defining the position of junctions or cut-off values of spline functions or formation functions over equally spaced pair values with 3 internal knot and defined the maximum lag range as 21 through literature review^22^. In this model α is the intercept; ns(.) means a natural cubic spline; β is the regression coefficient of DOW_t_; and t is the day of the week on day. We chose 1 df (degrees of freedom) for each year, 3 df for Ap, and 3df for Rh^23^. Then we plotted the exposure-lag-response diagram of temperature and in-hospital mortality by estimating the relative risk (RR) with 95% confidence interval (CI) of in-hospital dying on a day. When fitting the model, the RR of in-hospital death per day was lower at higher temperature. By comparison, 28 °C was chosen as the moderate temperature (MT) for the model (the higher 90% of daily temperatures). The temperature was divided into four grades, extreme cold (defined as ≤ 2.5th percentile of daily mean temperatures), moderate cold (define as 2.5th percentile of daily mean temperatures to the MT), moderate heat (define as MT to 97.5th percentile of daily mean temperatures), and extreme heat (≥ 97.5th percentile of daily mean temperatures).

The stratified analysis used in this study divided the suspected confounding factors into different levels, and then the association strength between exposure and disease was analyzed respectively in each level so that the influence of confounding factors on research results could be controlled to a certain extent^24,25^. We stratified age to < 65 years and ≥ 65 years and gender to males and females. Stratified analysis was further used to analyze the temperature effect between different age groups and gender. DLNM analyses were performed using the “dlnm” package of R (version 4.0.2 for Windows)^26^.

### Ethics approval and consent to participate

The study was conducted according to the Declaration of Helsinki. The study was approved by the Institutional Ethics Committee of Jiangsu Province Hospital (2020-QT-14) and individual consent for this retrospective analysis was waived.

## Results

Table 1 shows the summary statistics of the daily in-hospital deaths, average daily temperatures, average air pressure, and average daily relative humidity. This study includes 6160 deaths with 70.9% (4368) ≥ 65 years old and 67.0% (4127) male. The average daily mean temperature is 16.6 °C.

**Table 1:** Summary statistics of in-hospital deaths and climatic variables, 2010–2020.

	Mean ± SD	Min	P2.5	P25	P50	P75	P97.5	Max
**Daily in-hospital death (N)**	1.6 ± 1.5	0	0	0	1	2	5	12
< 65 year	0.5 ± 0.7	0	0	0	0	1	2	6
≥ 65 year	1.1 ± 1.2	0	0	0	1	2	4	10
Male	1.1 ± 1.2	0	0	0	1	2	4	8
Female	0.5 ± 0.8	0	0	0	0	1	2	8
Average mean temperature (Temp, °C)	16.6 ± 9.2	− 6.7	0.2	8.5	17.40	24.2	31.6	34.7
Average mean air pressure (Rh, %)	71.5 ± 14.5	17	42.0	62.0	72.0	82.5	96.0	100
Average mean relative humidity (Ap, kPa)	1012.4 ± 9.3	986.8	997.3	1004.5	1012.7	1019.7	1029.3	1038.8

**Figure 1:**
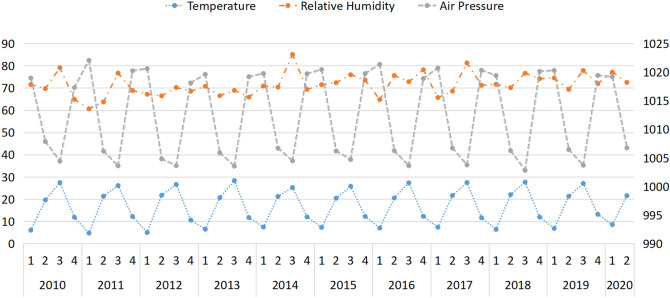
Trends in climatic factors according to quarter over the 10-year study period (2010–2020).

Figure 1 shows the trends of temperatures, relative humidity, and air pressure over quarter. We find that the seasonal trend of temperatures and pressure is the same in every year, but the seasonal trend of relative humidity is not obvious.

**Figure 2:**
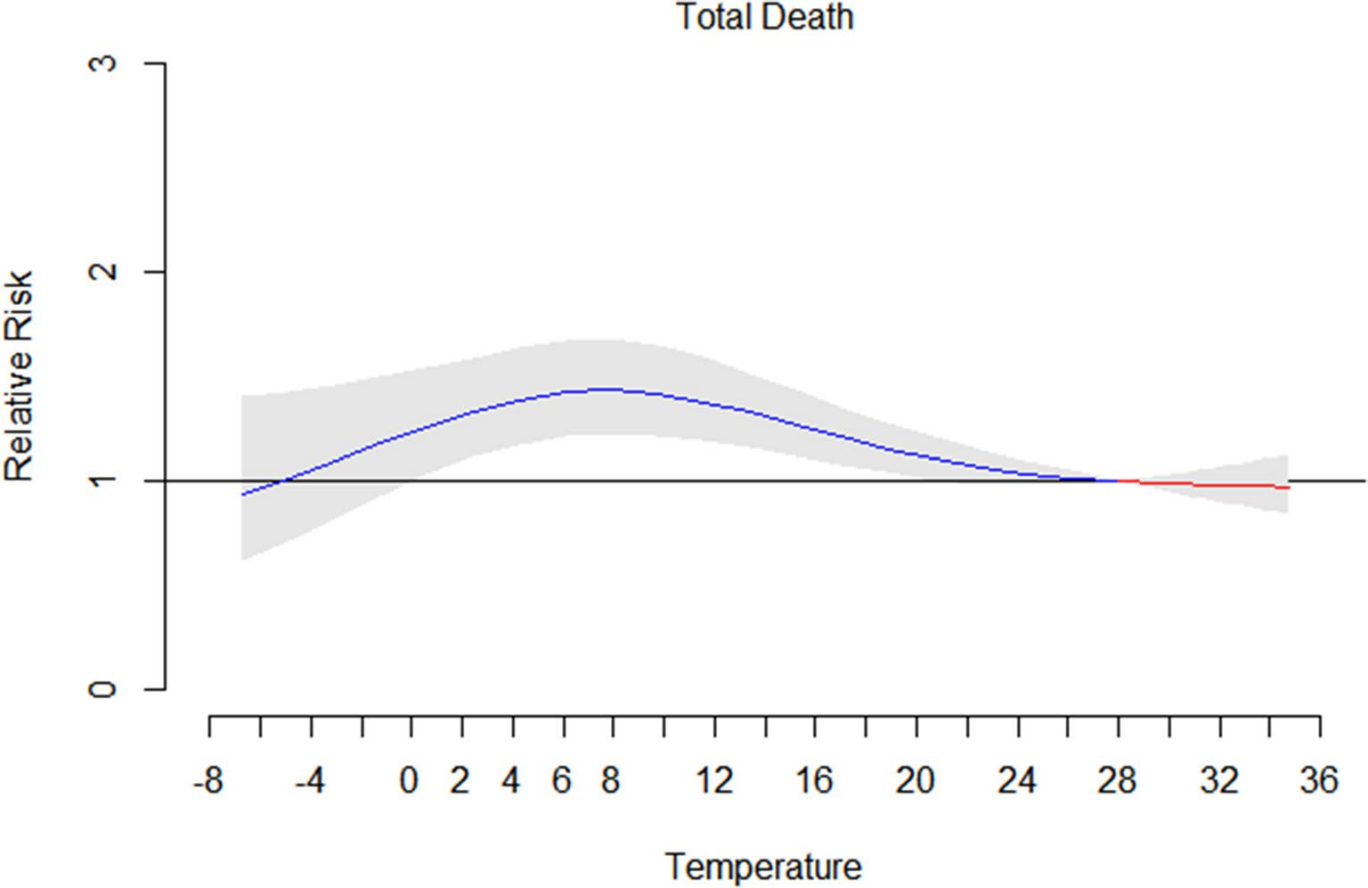
Estimated temperature effects (mean temperature, lag 0–21) by total in-hospital death, 2010–2020.

Figure 2 shows the relationships (lag 0–21 days) between daily mean temperatures and in-hospital death predicted by the DLNM, with 95% confidence interval (95% CI). It indicates the inverted V-shaped temperature-mortality relationships. Daily in-hospital deaths were first positive and then negative associated with daily mean temperatures. The peak of RR appeared at 8 °C with significant association (1.04, 95% CI 1.22–1.68). The RR with 95% CI between temperature 1–20 °C was higher than 1.0 (seen in Supplementary 1). Moderate cold related to higher RR value, we defined it as the moderate cold effect in this study.

**Figure 3:**
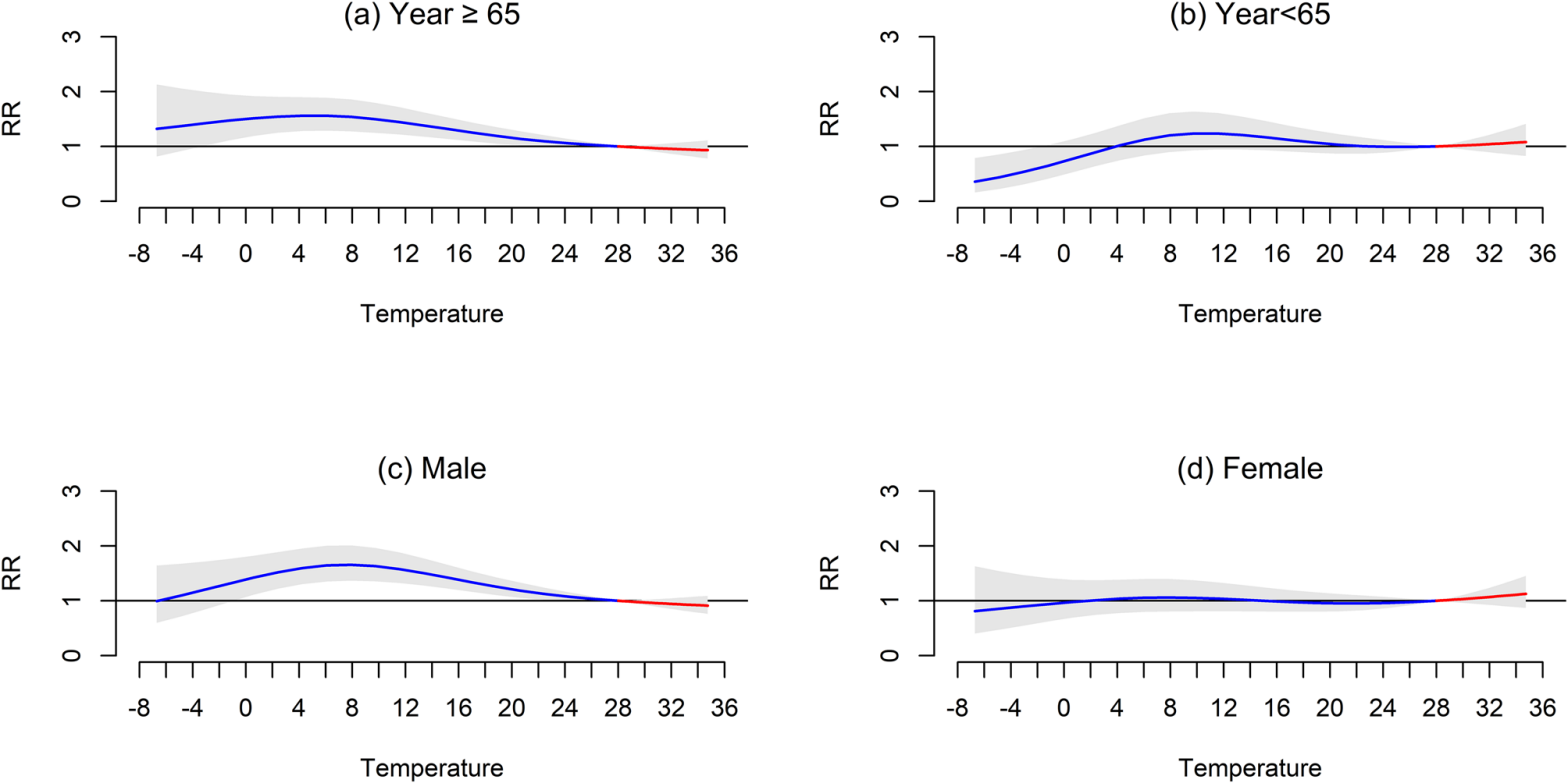
Stratified analysis temperature effects (mean temperature, lag 0–21 days) by total in-hospital death between age groups and gender, 2010–2020.

Stratified analysis between age (< 65 years and ≥ 65 years groups) and gender (male and female) also showed the moderate cold effect (seen in Supplementary 1). The effect ≥ 65 years in Fig. 3a was more pronounced to < 65-year-old in Fig. 3b. RR with 95% CI > 1.0 was found between − 3 to 21 °C in group ≥ 65 years but was not found in < 65 years. The peak of RR appeared at 5 °C with a significant association in group ≥ 65 years (1.56, 95% CI 1.27–1.90), and 11 °C with no association in group < 65 years (1.27, 95% CI 0.94–1.62). The moderate cold effect on death was different between genders. RR with 95% CI > 1.0 was found between 0 to 24 °C in males but not in females (Fig. 3c,d). The peak of RR appeared at 8 °C with a significant association in males (1.66, 95% CI 1.37–2.01), and 8 °C with no association in females (1.06, 95% CI 0.81–1.40).

**Figure 4:**
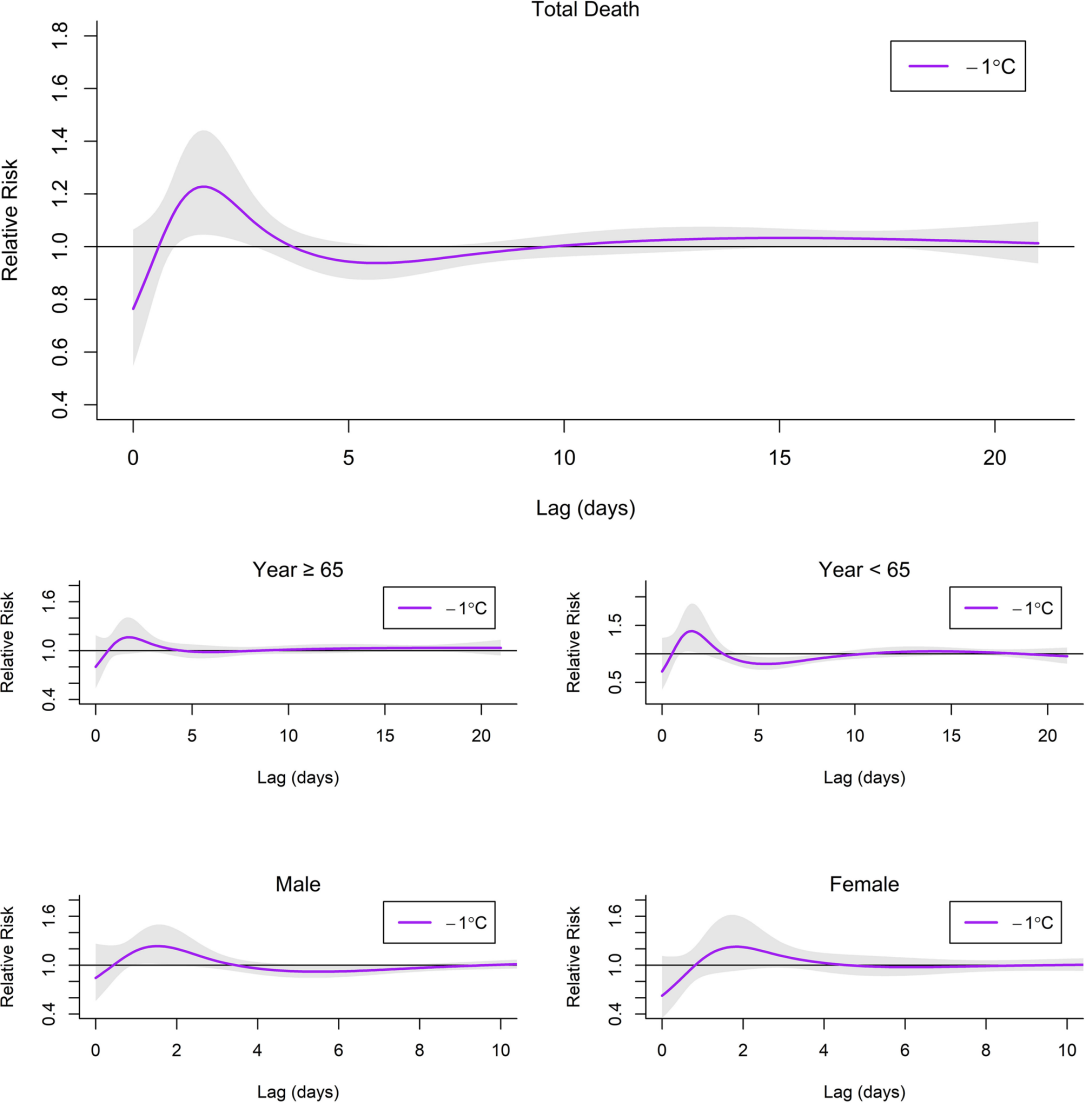
The estimated relative risk of dying on a day with − 1 °C compared with that on a day with 28 °C (MT) over 21 lagged days for all summers (whole study period).

Furthermore, we plot the estimated lag-response curve with a − 1 °C (Fig. 4). The RR of in-hospital mortality was lower than 1.0 on first day (lag 0: RR (Total) = 0.76, 95% CI 0.55–1.06, RR (≥ 65) = 0.80, 95% CI 0.54–1.19, RR (< 65) = 0.69, 95% CI 0.37–1.28, RR (male) = 0.84, 95% CI 0.56–1.26, RR (female) = 0.62, 95% CI 0.35–1.11). Then sharply increased in the next 2 days and finally levelled off. The RR peak of in-hospital mortality was at lag 1.6 (1.23, 95% CI 1.05, 1.44) for total mortality, lag 1.7 (1.16, 95% CI 0.96–1.41) for ≥ 65 years, lag 1.5 (1.40, 95% CI 1.04–1.88) for < 65 years, lag 1.0 (1.72, 95% CI 1.16–2.55) for male and lag 1.1 (1.79, 95% CI 1.02–3.19) for female.

## Discussion

There have been many similar studies in the general population in the past, but they have been of little use in guiding the management of a focus group such as hospitalized patients^1,7,11^. As far as we know, there was no study on the relationship between mortality risk and ambient temperatures among in-hospital patients. This study is more personalized and focuses on key groups, taking inpatients as the research target, which provides an important scientific basis for the management of inpatients. We explored the association of ambient temperatures and in-hospital mortality in a large research hospital, located in Nanjing, Jiangsu, China, using 10 years mortality data during 2010–2020 by DLNM analyses.

Normal physiologic Responses of healthy bodies respond to moderate high and low temperatures by sensing changes in skin and core temperatures. For heat, which would cause blood vessel dilation (vasodilation), a significant increase in pumping rate (cardiac output), and sweating. Blood pressure drops in warm temperatures due to vasodilation and dehydration^27–29^. By contrast, in cold temperatures, blood vessels narrow (vasoconstriction), slowing the process of transferring heat to the surface of the body^30^. Enhancement or impairment of any organ system (nervous, endocrine, renal, cardiovascular, or cutaneous) involved in thermoregulation alters sensitivity to high and low temperatures. Liu et al. reported that temperature-induced injury is thought to be associated with the sympathetic nervous system, enhanced sympathetic response to renin-angiotensin system activation, dehydration, and systemic inflammatory responses^31^. It has been reported that high temperatures may disrupt sleep, with one study in Detroit finding that blood pressure rose in the morning after a hot night^32^. Blood viscosity, cholesterol, and platelets had a seasonal pattern with a peak time in winter, which may increase the risk of a heart attack or stroke^33^. However, Song et al. reported that cold or heat waves that occur early in the cool or warm season may be more dangerous because of accumulation of susceptibility pools or lack of preparation for extreme temperatures, which is similar to our results^34^.

In this study, we found that moderate cold had an impact on the increased risk of in-hospital mortality. The temperature at the lowest death rate was 28 °C in the study, which was in 90% of all temperatures. The difference is that previous studies have reported that heat exposure is a health threat. A meta-analysis found that heat exposure was associated with increased risk of cardiovascular, cerebrovascular, and respiratory mortality^35^. Lu et al. indicated that the burden of cardiovascular hospitalizations caused by high temperature could increase in the context of global warming^36^. Consistent with this study, many studies indicated that while the burden of temperature-related mortality may shift to higher temperature in the future, cold temperature may be a bigger problem in temperate cities today^27,37,38^. A review reported that deaths and hospitalizations due to extreme heat increased sharply in the Detroit area, while deaths due to cold temperatures increased gradually^27^. A study conducted in Hong Kong noted that low temperatures had a greater impact on non-accidental, cardiovascular, respiratory, and cancer deaths than high temperatures^38^.

Furthermore, we found that moderate cold, not extreme temperatures, had the highest risk for mortality. These results were similar to some previous studies. Research in China had reported that cold temperatures was responsible for a higher proportion of deaths than heat^39^. Gasparrini et al. reported that the effect of days with extreme temperatures was less than milder but non-optimum weather not only in China but in other countries^1^. It has also been demonstrated that moderate cold (2.5th percentile to the MMT) attribute higher percent (6.66%) of mortality than extreme cold (0.63%) and extreme heat (0.23%)^40^. Another research in China had similar results that 1.14%, 10.49%, 2.08%, and 0.63% of the mortality were attributable to extreme cold (− 6.4 to − 1.4 °C), moderate cold (− 1.4 to 22.8 °C), moderate heat (22.8 to 29 °C), and extreme heat (29.0 to 31.6 °C), respectively^2^.

This finding may be explained by three reasons. First, compared with these places, Jiangsu Province had a temperate monsoon climate with fewer extreme weather^16^. Hence, the effect of extreme temperatures on mortality may be reduced. This was similar to Antonio Gasparrini's study that the temperatures percentile of minimum mortality varied from roughly the 60th percentile in tropical areas to about the 80–90th percentile in temperate regions^1^. Second, the effects of air conditioning can weaken the effect of extreme heat and cold but can highlight the risk of moderate cold. All the wards in the hospital are equipped with air conditioners. Whether the temperatures is high or low in summer and winter, the hospital will turn on the air conditioning. As a result, there is almost no extreme weather for hospitalized patients. However, hospital air conditioners are often turned off in mild temperatures, and it is more likely to produce negative effects on health for the inadaptation of inpatients. Although Alberini et al. reported that air conditioning ownership was not associated with self-reported heat illness in a study in Canada^41^. More reports suggest that air conditioning plays a positive role in the link between temperatures and mortality^42–46^. Deng et al. provided evidence that daily mean temperatures and DTR were significantly associated with non-accidental mortality and had delayed effects^13^. Third, Jiangsu province has a high level of economic development with the top three highest GDP in China, which is less susceptible to extreme temperatures^47,48^. The governments in high-GDP regions can build quality infrastructure and have a larger capacity to cope with extreme temperatures. On the contrary, governments in low-GDP areas have fewer resources for preventative and adaptive measures and lack the resources to cope with the effects of extreme temperatures^47^.

There are many factors leading to whether an individual is susceptible to temperatures, including medications and alcohol, homelessness, age, and so on^29^. Anticholinergic, antihypertensive, and antipsychotic drugs, used to treat disorders of the nervous, endocrine, renal, or cardiovascular systems, may impair thermoregulation which caused the decrease of individual's ability to sense heat or cold, or may inhibit other temperature-regulating responses^49^. Qualitative studies in Detroit confirmed the findings of qualitative and survey studies in other cities that cost is a major barrier to the use of air conditioning^50,51^. It also has been found that income or poverty is associated with heat-related mortality at the community level in the United States, China, and Japan^52–54^.With age, even in the absence of obvious heart disease and heart failure, the amount of blood pumped per heartbeat (medium air volume) decreases, as does the ability of blood vessels to dilate and contract^55,56^. At the same time, the loss of muscle mass also leads to a reduction in internal heat production, although increased fat storage retains heat^57^.

In this study we explore the relationship between in-hospital mortality and temperatures in patients of different genders and ages, we performed a stratified analysis for different age and sex subgroups in our study. We found that older patients (≥ 65 years) and males were more susceptible to moderate cold in in-hospital mortality, and have a longer range of risk temperatures. Current literature on the effect of temperatures on age are mostly consistent, that elderly people are more like be influenced by temperatures^58,59^. Park et al. found the mortality rates of elderly outdoor workers increased consistently with temperatures^59^. In addition, we found elderly patients had a narrower risk temperatures range (− 3 to 21 °C) than total patients (1 to 20 °C). This can verify the above results.

The effect of gender on the relationship between mortality and temperatures was conflicting. Some researchers reported that males were more vulnerable than females to temperatures^60–62^, which was consistent with this study. Junkka et al. reported that the OR of mortality at − 20 °C was 1.17 (0.88–1.54) among females, and 1.94 (1.53–2.45) among males^62^. Zhai et al. reported that the cold temperatures effect of males was stronger than that of females, and the number of death for males was 118,186 and for females was 111,002^63^. But there also had some studies with no differences between males and females. Basu et al. reported that no significant difference in mortality was found between males (2.8%, 95% CI 1.1, 4.6) and females (2.6%, 95% CI 1.2, 3.9)^64^. In addition, other studies confirmed that diurnal temperature range threatening to vulnerable groups, and females and elderly mortality^65^. Deng et al. also pointed out that females were more susceptible to high diurnal temperature range effect than males with 17.01, 11.82 death per day, respectively^13^.

In addition, there were also several limitations that should be addressed in this study. The temperature used in this study is the ambient temperature, not the actual temperature situation around the patient. The actual temperatures of the environment patients live can better reflect the health effects of temperatures. More studies should be conducted on the relationship between ambient temperatures around patients and in-hospital mortality. We did not analyze the kind of mortality in this study for the unavailable of data. In addition, although this hospital is the largest general hospital in Jiangsu Province, these results are still limited to some extent. Therefore, more relevant data from more hospitals should be collected in the future.

## Conclusion

This study found a correlation between average temperature and inpatient deaths and was influenced by gender and age. Moderate cold temperatures was an increased risk for in-hospital death, with the elderly (≥ 65 years) and male patients being more sensitive to the effects of moderate cold. The results of this study need to be further confirmed in other hospitals, but it still has reference significance for the management of other hospitals and the reduction of hospital mortality. This result shows the importance of moderate temperatures for health, and hospital managers should pay more attention to patients during moderate cold temperatures. During periods of moderate cold, patient protection should be increased, such as personalized air conditioning and additional clothing and bedding. In addition, attention should be paid to older elderly and female patients in hospitals. In terms of the hospital environment and planning design, attention should be paid to the use and management of air conditioning, and strengthen the temperature monitoring of wards.

For the public health department, this evidence also has important implications to the planning of interventions to decrease the health risk of harmful temperatures in the hospital. It also suggests that health authorities cannot ignore the risk of moderate cold. In moderate cold weather, health administrative departments should strengthen temperature monitoring and guide hospital managers to adopt personalized temperature management programs such as air conditioning management in weather changes.

In addition, this study also suggests that we should pay more attention to the relationship between special groups and environment in the follow-up research in the future.

## Supplementary Information

Supplementary Table 1.

## Data Availability

The datasets used and/or analyzed during the current study are available from the corresponding author on reasonable request.

## References

[1] Gasparrini A, Guo Y, Hashizume M (2015). Mortality risk attributable to high and low ambient temperature: A multicountry observational study. Lancet.

[2] Chen R, Yin P, Wang L (2018). Association between ambient temperature and mortality risk and burden: Time series study in 272 main Chinese cities. BMJ.

[3] Sheridan S-C, Lee C-C, Allen M-J (2019). The mortality response to absolute and relative temperature extremes. Int. J. Environ. Res. Public Health.

[4] Chen T-H, Du XL, Chan W (2019). Impacts of cold weather on emergency hospital admission in Texas, 2004–2013. Environ. Res..

[5] Luo Y, Li H, Huang F (2018). The cold effect of ambient temperature on ischemic and hemorrhagic stroke hospital admissions: A large database study in Beijing, China between years 2013 and 2014—Utilizing a distributed lag non-linear analysis. Environ. Pollut..

[6] Ha J, Kim H, Hajat S (2011). Effect of previous-winter mortality on the association between summer temperature and mortality in South Korea. Environ. Health Perspect..

[7] Kim H, Ha J-S, Park J (2006). High temperature, heat index, and mortality in 6 major cities in South Korea. Arch. Environ. Occup. Health.

[8] Lim Y-H, Kim H, Kim J-H (2013). Effect of diurnal temperature range on cardiovascular markers in the elderly in Seoul, Korea. Int. J. Biometeorol..

[9] Son J-Y, Bell M-L, Lee J-T (2014). The impact of heat, cold, and heat waves on hospital admissions in eight cities in Korea. Int. J. Biometeorol..

[10] Su X, Cheng Y, Wang Y (2019). Regional temperature-sensitive diseases and attributable fractions in China. Int. J. Environ. Res. Public Health.

[11] Ma W, Wang L, Lin H (2015). The temperature-mortality relationship in China: An analysis from 66 Chinese communities. Environ. Res..

[12] Dai Q, Ma W, Huang H (2018). The effect of ambient temperature on the activity of influenza and influenza like illness in Jiangsu Province, China. Sci. Total Environ..

[13] Deng J, Hu X, Xiao C (2020). Ambient temperature and non-accidental mortality: A time series study. Environ. Sci. Pollut. Res. Int..

[14] Gasparrini A (2011). Distributed lag linear and non-linear models in R: The package dlnm. J. Stat. Softw..

[15] Nanjing has a permanent population of 9.4234 million by the end of 2021_Dynamic work_Nanjing Bureau of Statistics, 2022/2/16. http://tjj.nanjing.gov.cn/gzdt/202202/t20220215_3293790.html

[16] Nanjing Reference materials, 2021/10/15. https://baike.baidu.com/reference/23952/46d3UjJiCpmhLd2z4ZOqZ7_oofA4u0FK6_cAV1wlyNVdxYlmQDXeh1NuOivfIVneg2pD33cwhyRI5pXT1d1SKkHikuzsQZRXkyI-rvy-UsR0-6l85zOLL7paIlqqVQ

[17] National Meteorological Information Center—China Meteorological Data Network, 2021/10/15. http://data.cma.cn/

[18] Exploration on high-quality development of Jiangsu Provincial People's Hospital, 2021/10/15. https://mp.weixin.qq.com/s/URaSzszbn7IG5PwlpH8H9A

[19] Brief introduction of Jiangsu Provincial People's Hospital (The First Affiliated Hospital of Nanjing Medical University), 2021/10/15. http://www.jsph.org.cn/yiyuangaikuang/jinrishengyi/

[20] Gasparrini A, Leone M (2014). Attributable risk from distributed lag models. BMC Med. Res. Methodol..

[21] Gasparrini A, Armstrong B, Kenward M-G (2010). Distributed lag non-linear models. Stat. Med..

[22] Guo Y, Gasparrini A, Armstrong B (2014). Global variation in the effects of ambient temperature on mortality: A systematic evaluation. Epidemiology.

[23] Bhaskaran K, Gasparrini A, Hajat S (2013). Time series regression studies in environmental epidemiology. Int. J. Epidemiol..

[24] Miller K, Carles J, Gschwend J-E (2018). The phase 3 COU-AA-302 study of abiraterone acetate plus prednisone in men with chemotherapy-naive metastatic castration-resistant prostate cancer: Stratified analysis based on pain, prostate-specific antigen, and Gleason score. Eur. Urol..

[25] Petersen T, Christensen R, Juhl C (2015). Predicting a clinically important outcome in patients with low back pain following McKenzie therapy or spinal manipulation: A stratified analysis in a randomized controlled trial. BMC Musculoskelet. Disord..

[26] R: The R Project for Statistical Computing, 2021/10/15. https://www.r-project.org/

[27] Gronlund C-J, Sullivan K-P, Kefelegn Y (2018). Climate change and temperature extremes: A review of heat- and cold-related morbidity and mortality concerns of municipalities. Maturitas.

[28] Kenny G-P, Yardley J, Brown C (2010). Heat stress in older individuals and patients with common chronic diseases. CMAJ.

[29] Crandall C-G, Gonzalez-Alonso J (2010). Cardiovascular function in the heat-stressed human. Acta Physiol. (Oxf).

[30] Castellani J-W, Young A-J (2016). Human physiological responses to cold exposure: Acute responses and acclimatization to prolonged exposure. Auton. Neurosci..

[31] Liu C, Yavar Z, Sun Q (2015). Cardiovascular response to thermoregulatory challenges. Am. J. Physiol. Heart Circ. Physiol..

[32] Brook R-D, Shin H-H, Bard R-L (2011). Can personal exposures to higher nighttime and early-morning temperatures increase blood pressure?. J. Clin. Hypertens. (Greenwich).

[33] Hopstock L-A, Barnett A-G, Bonaa K-H (2013). Seasonal variation in cardiovascular disease risk factors in a subarctic population: The Tromso Study 1979–2008. J. Epidemiol. Community Health.

[34] Barnett A-G, Hajat S, Gasparrini A (2012). Cold and heat waves in the United States. Environ. Res..

[35] Song X, Wang S, Hu Y (2017). Impact of ambient temperature on morbidity and mortality: An overview of reviews. Sci. Total Environ..

[36] Lu P, Xia G, Zhao Q (2020). Temporal trends of the association between ambient temperature and hospitalisations for cardiovascular diseases in Queensland, Australia from 1995 to 2016: A time-stratified case-crossover study. PLoS Med..

[37] Huber V, Krummenauer L, Pena-Ortiz C (2020). Temperature-related excess mortality in German cities at 2 °C and higher degrees of global warming. Environ. Res..

[38] Liu S, Chan EYY, Goggins W-B (2020). The mortality risk and socioeconomic vulnerability associated with high and low temperature in Hong Kong. Int. J. Environ. Res. Public Health.

[39] Zhang Y, Wang S, Zhang X (2020). Association between moderately cold temperature and mortality in China. Environ. Sci. Pollut. Res. Int..

[40] Gasparrini A, Guo Y, Hashizume M (2015). Temporal variation in heat-mortality associations: A multicountry study. Environ. Health Perspect..

[41] Alberini A, Gans W, Alhassan M (2011). Individual and public-program adaptation: Coping with heat waves in five cities in Canada. Int. J. Environ. Res. Public Health.

[42] Anderson B-G, Bell M-L (2009). Weather-related mortality: How heat, cold, and heat waves affect mortality in the United States. Epidemiology.

[43] O'Neill M-S, Zanobetti A, Schwartz J (2005). Disparities by race in heat-related mortality in four US cities: The role of air conditioning prevalence. J. Urban Health.

[44] Braga A-L, Zanobetti A, Schwartz J (2001). The time course of weather-related deaths. Epidemiology.

[45] Ostro B, Rauch S, Green R (2010). The effects of temperature and use of air conditioning on hospitalizations. Am. J. Epidemiol..

[46] Medina-Ramon M, Schwartz J (2007). Temperature, temperature extremes, and mortality: A study of acclimatisation and effect modification in 50 US cities. Occup. Environ. Med..

[47] Yang Z, Wang Q, Liu P (2019). Extreme temperature and mortality: Evidence from China. Int. J. Biometeorol..

[48] China's provincial GDP ranking 2020 Complete edition (2020 Provincial GDP Ranking), 2021/10/15. http://www.cwtea.net/article/18137.html

[49] Gronlund C-J, Berrocal V-J, White-Newsome J-L (2015). Vulnerability to extreme heat by socio-demographic characteristics and area green space among the elderly in Michigan, 1990–2007. Environ. Res..

[50] Gronlund C-J (2014). Racial and socioeconomic disparities in heat-related health effects and their mechanisms: A review. Curr. Epidemiol. Rep..

[51] Sampson N-R, Gronlund C-J, Buxton M-A (2013). Staying cool in a changing climate: Reaching vulnerable populations during heat events. Glob. Environ. Change.

[52] Madrigano J, Mittleman M-A, Baccarelli A (2013). Temperature, myocardial infarction, and mortality: Effect modification by individual- and area-level characteristics. Epidemiology.

[53] Chan E-Y, Goggins W-B, Kim J-J (2012). A study of intracity variation of temperature-related mortality and socioeconomic status among the Chinese population in Hong Kong. J. Epidemiol. Community Health.

[54] Ng C-F, Ueda K, Takeuchi A (2014). Sociogeographic variation in the effects of heat and cold on daily mortality in Japan. J. Epidemiol..

[55] Charkoudian N (2010). Mechanisms and modifiers of reflex induced cutaneous vasodilation and vasoconstriction in humans. J. Appl. Physiol..

[56] Kenney W-L, Craighead D-H, Alexander L-M (2014). Heat waves, aging, and human cardiovascular health. Med. Sci. Sports Exerc..

[57] Kenney W-L, Munce T-A (2003). Invited review: Aging and human temperature regulation. J. Appl. Physiol..

[58] Xing Q, Sun Z, Tao Y (2020). Impacts of urbanization on the temperature-cardiovascular mortality relationship in Beijing, China. Environ. Res..

[59] Park J, Chae Y, Choi S-H (2019). Analysis of mortality change rate from temperature in summer by age, occupation, household type, and chronic diseases in 229 Korean municipalities from 2007–2016. Int. J. Environ. Res. Public Health.

[60] Bell M-L, O'Neill M-S, Ranjit N (2008). Vulnerability to heat-related mortality in Latin America: A case-crossover study in Sao Paulo, Brazil, Santiago, Chile and Mexico City, Mexico. Int. J. Epidemiol..

[61] Bai L, Cirendunzhu, Woodward A (2014). Temperature and mortality on the roof of the world: A time-series analysis in three Tibetan counties, China. Sci. Total Environ..

[62] Junkka J, Karlsson L, Lundevaller E (2021). Climate vulnerability of Swedish newborns: Gender differences and time trends of temperature-related neonatal mortality, 1880–1950. Environ. Res..

[63] Zhai L, Ma X, Wang J (2022). Effects of ambient temperature on cardiovascular disease: A time-series analysis of 229288 deaths during 2009–2017 in Qingdao, China. Int. J. Environ. Health Res..

[64] Basu R, Ostro B-D (2008). A multicounty analysis identifying the populations vulnerable to mortality associated with high ambient temperature in California. Am. J. Epidemiol..

[65] Zhao Y-Q, Wang L-J, Luo Y (2017). Lagged effects of diurnal temperature range on mortality in 66 cities in China: A time-series study. Zhonghua Liu Xing Bing Xue Za Zhi.

