# Supplementary material for: The effect of ambient temperature on in-hospital mortality: a study in Nanjing, China
Source: Sci Rep. 2022 Apr 15;12:6304. doi: 10.1038/s41598-022-10395-6 (PMC9012784; doi:10.1038/s41598-022-10395-6)
Supplement: Supplementary file 1 — Supplementary Table 1. [file 41598_2022_10395_MOESM1_ESM.docx]

Supplementary 1. The RR (95%CI) for total and subgroups mortality and corresponding temperature.

| Temperature (℃ ) | Total Death | ≥65 year | <65 | Male | Female |
| --- | --- | --- | --- | --- | --- |
| -7 | 0.92 (0.61, 1.40) | 1.31 (0.80, 2.14) | 0.35 (0.15, 0.78) | 0.98 (0.58, 1.64) | 0.80 (0.39, 1.64) |
| -6 | 0.96 (0.66, 1.41) | 1.34 (0.86, 2.10) | 0.39 (0.18, 0.81) | 1.03 (0.64, 1.65) | 0.83 (0.43, 1.59) |
| -5 | 1.01 (0.71, 1.43) | 1.37 (0.91, 2.06) | 0.43 (0.22, 0.85) | 1.09 (0.71, 1.67) | 0.85 (0.47, 1.55) |
| -4 | 1.05 (0.77, 1.44) | 1.40 (0.96, 2.03) | 0.48 (0.26, 0.89) | 1.15 (0.78, 1.69) | 0.87 (0.51, 1.50) |
| -3 | 1.10 (0.82, 1.46) | 1.42 (1.02, 1.99) | 0.54 (0.31, 0.93) | 1.21 (0.85, 1.72) | 0.90 (0.55, 1.46) |
| -2 | 1.14 (0.88, 1.48) | 1.45 (1.07, 1.97) | 0.60 (0.36, 0.98) | 1.27 (0.92, 1.74) | 0.92 (0.59, 1.43) |
| -1 | 1.19 (0.94, 1.50) | 1.48 (1.12, 1.94) | 0.66 (0.42, 1.03) | 1.33 (1.00, 1.77) | 0.94 (0.63, 1.41) |
| 0 | 1.23 (1.00, 1.52) | 1.50 (1.17, 1.93) | 0.73 (0.49, 1.09) | 1.39 (1.07, 1.80) | 0.96 (0.67, 1.39) |
| 1 | 1.28 (1.05, 1.55) | 1.52 (1.21, 1.91) | 0.80 (0.55, 1.15) | 1.44 (1.14, 1.83) | 0.98 (0.70, 1.38) |
| 2 | 1.31 (1.10, 1.58) | 1.54 (1.24, 1.90) | 0.87 (0.62, 1.22) | 1.50 (1.20, 1.87) | 1.00 (0.73, 1.37) |
| 3 | 1.35 (1.14, 1.60) | 1.55 (1.26, 1.90) | 0.94 (0.68, 1.29) | 1.55 (1.25, 1.91) | 1.02 (0.76, 1.37) |
| 4 | 1.38 (1.17, 1.63) | 1.56 (1.28, 1.90) | 1.00 (0.74, 1.37) | 1.59 (1.29, 1.94) | 1.03 (0.78, 1.38) |
| 5 | 1.40 (1.19, 1.65) | 1.56 (1.29, 1.90) | 1.07 (0.79, 1.44) | 1.62 (1.33, 1.98) | 1.05 (0.79, 1.39) |
| 6 | 1.42 (1.21, 1.67) | 1.56 (1.29, 1.89) | 1.12 (0.83, 1.51) | 1.64 (1.35, 2.00) | 1.05 (0.80, 1.40) |
| 7 | 1.43 (1.22, 1.68) | 1.55 (1.28, 1.88) | 1.17 (0.87, 1.57) | 1.65 (1.36, 2.01) | 1.06 (0.80, 1.40) |
| 8 | 1.43 (1.22, 1.68) | 1.54 (1.27, 1.85) | 1.20 (0.90, 1.61) | 1.66 (1.37, 2.01) | 1.06 (0.81, 1.40) |
| 9 | 1.42 (1.22, 1.66) | 1.52 (1.26, 1.82) | 1.23 (0.92, 1.63) | 1.64 (1.36, 1.99) | 1.06 (0.81, 1.39) |
| 10 | 1.41 (1.21, 1.64) | 1.49 (1.25, 1.78) | 1.24 (0.94, 1.63) | 1.62 (1.35, 1.95) | 1.05 (0.81, 1.37) |
| 11 | 1.39 (1.20, 1.61) | 1.46 (1.23, 1.74) | 1.24 (0.94, 1.62) | 1.60 (1.33, 1.91) | 1.04 (0.81, 1.35) |
| 12 | 1.37 (1.19, 1.57) | 1.43 (1.21, 1.69) | 1.23 (0.95, 1.60) | 1.56 (1.31, 1.85) | 1.03 (0.81, 1.32) |
| 13 | 1.34 (1.17, 1.53) | 1.40 (1.19, 1.64) | 1.21 (0.95, 1.56) | 1.52 (1.29, 1.79) | 1.02 (0.81, 1.30) |
| 14 | 1.31 (1.15, 1.49) | 1.36 (1.16, 1.59) | 1.19 (0.94, 1.52) | 1.48 (1.26, 1.73) | 1.01 (0.81, 1.27) |
| 15 | 1.27 (1.13, 1.44) | 1.32 (1.14, 1.54) | 1.17 (0.93, 1.47) | 1.43 (1.23, 1.66) | 1.00 (0.80, 1.24) |
| 16 | 1.24 (1.10, 1.40) | 1.29 (1.12, 1.48) | 1.14 (0.92, 1.42) | 1.38 (1.20, 1.60) | 0.99 (0.80, 1.22) |
| 17 | 1.21 (1.08, 1.36) | 1.25 (1.09, 1.44) | 1.12 (0.91, 1.37) | 1.34 (1.16, 1.54) | 0.98 (0.80, 1.19) |
| 18 | 1.18 (1.06, 1.31) | 1.22 (1.07, 1.39) | 1.09 (0.89, 1.33) | 1.29 (1.13, 1.47) | 0.97 (0.80, 1.17) |
| 19 | 1.15 (1.03, 1.27) | 1.19 (1.05, 1.34) | 1.06 (0.88, 1.29) | 1.25 (1.10, 1.42) | 0.96 (0.80, 1.15) |
| 20 | 1.12 (1.02, 1.24) | 1.16 (1.03, 1.30) | 1.04 (0.87, 1.25) | 1.21 (1.07, 1.36) | 0.95 (0.80, 1.14) |
| 21 | 1.10 (1.00, 1.20) | 1.13 (1.01, 1.26) | 1.02 (0.87, 1.21) | 1.17 (1.05, 1.31) | 0.95 (0.81, 1.12) |
| 22 | 1.08 (0.99, 1.17) | 1.10 (1.00, 1.22) | 1.01 (0.87, 1.18) | 1.14 (1.03, 1.26) | 0.95 (0.82, 1.10) |
| 23 | 1.06 (0.98, 1.14) | 1.08 (0.99, 1.18) | 1.00 (0.87, 1.15) | 1.11 (1.01, 1.21) | 0.95 (0.84, 1.09) |
| 24 | 1.04 (0.98, 1.11) | 1.06 (0.98, 1.15) | 0.99 (0.89, 1.11) | 1.08 (1.00, 1.17) | 0.96 (0.86, 1.07) |
| 25 | 1.03 (0.98, 1.08) | 1.04 (0.98, 1.11) | 0.99 (0.91, 1.09) | 1.06 (1.00, 1.12) | 0.97 (0.89, 1.05) |
| 26 | 1.02 (0.98, 1.05) | 1.03 (0.99, 1.07) | 0.99 (0.93, 1.06) | 1.04 (0.99, 1.08) | 0.98 (0.92, 1.04) |
| 27 | 1.01 (0.99, 1.03) | 1.01 (0.99, 1.04) | 0.99 (0.96, 1.03) | 1.02 (1.00, 1.04) | 0.99 (0.96, 1.02) |
| 28 | 1.00 (1.00, 1.00) | 1.00 (1.00, 1.00) | 1.00 (1.00, 1.00) | 1.00 (1.00, 1.00) | 1.00 (1.00, 1.00) |
| 29 | 0.99 (0.97, 1.01) | 0.99 (0.97, 1.01) | 1.01 (0.97, 1.04) | 0.98 (0.96, 1.01) | 1.02 (0.98, 1.05) |
| 30 | 0.99 (0.95, 1.03) | 0.98 (0.93, 1.02) | 1.02 (0.95, 1.09) | 0.97 (0.92, 1.02) | 1.03 (0.96, 1.11) |
| 31 | 0.98 (0.93, 1.05) | 0.97 (0.90, 1.04) | 1.03 (0.92, 1.15) | 0.96 (0.89, 1.03) | 1.05 (0.94, 1.17) |
| 32 | 0.98 (0.90, 1.07) | 0.96 (0.87, 1.06) | 1.04 (0.89, 1.21) | 0.94 (0.85, 1.04) | 1.07 (0.92, 1.24) |
| 33 | 0.98 (0.88, 1.09) | 0.95 (0.83, 1.08) | 1.05 (0.87, 1.28) | 0.93 (0.82, 1.06) | 1.09 (0.90, 1.31) |
| 34 | 0.97 (0.86, 1.11) | 0.94 (0.80, 1.10) | 1.07 (0.84, 1.35) | 0.92 (0.78, 1.08) | 1.11 (0.89, 1.39) |
| 35 | 0.97 (0.83, 1.13) | 0.93 (0.77, 1.12) | 1.08 (0.82, 1.43) | 0.91 (0.75, 1.09) | 1.13 (0.87, 1.48) |
| 36 | 0.97 (0.81, 1.16) | 0.92 (0.75, 1.14) | 1.10 (0.80, 1.52) | 0.90 (0.72, 1.11) | 1.15 (0.85, 1.57) |
